# Association of clinical outcomes and the predictive value of T lymphocyte subsets within colorectal cancer patients

**DOI:** 10.3389/fsurg.2023.1102545

**Published:** 2023-05-03

**Authors:** Chaofeng Yuan, Jiannan Huang, Haitao Li, Rongnan Zhai, Jinjing Zhai, Xuedong Fang, Yuanyu Wu

**Affiliations:** ^1^ Department of Gastrointestinal Colorectal Surgery, China-Japan Union Hospital of Jilin University, Changchun, China; ^2^ Department of Orthopedics, China-Japan Union Hospital of Jilin University, Changchun, China

**Keywords:** colorectal cancer, flow cytometry, laparoscopy, tumor markers, immunity

## Abstract

**Introduction:**

Tumor immunity is a hot topic in tumor research today, and human immunity is closely related to tumor progression. T lymphocyte is an important component of human immune system, and the changes in their subsets may influence the progression of colorectal cancer (CRC) to some extent. This clinical study systematically describes and analyzes the association of CD4^+^ and CD8^+^ T-lymphocyte content and CD4^+^/CD8^+^ T-lymphocyte ratio with CRC differentiation, clinical pathological stage, Ki67 expression, T-stage, N-stage, carcinoembryonic antigen (CEA) content, nerve and vascular infiltration, and other clinical features, as well as preoperative and postoperative trends. Furthermore, a predictive model is constructed to evaluate the predictive value of T-lymphocyte subsets for CRC clinical features.

**Methods:**

Strict inclusion and exclusion criterion were formulated to screen patients, preoperative and postoperative flow cytometry and postoperative pathology reports from standard laparoscopic surgery were assessed. PASS and SPSS software, R packages were invoked to calculate and analyze.

**Results:**

We found that a high CD4^+^ T-lymphocyte content in peripheral blood and a high CD4^+^/CD8^+^ ratio were associated with better tumor differentiation, an earlier clinical pathological stage, lower Ki67 expression, shallower tumor infiltration, a smaller number of lymph node metastases, a lower CEA content, and a lower likelihood of nerve or vascular infiltration (*P* < 0.05). However, a high CD8^+^ T-lymphocyte content indicated an unpromising clinical profile. After effective surgical treatment, the CD4^+^ T-lymphocyte content and CD4^+^/CD8^+^ ratio increased significantly (*P* < 0.05), while the CD8^+^ T-lymphocyte content decreased significantly (*P* < 0.05). Further, we comprehensively compared the merits of CD4^+^ T-lymphocyte content, CD8^+^ T-lymphocyte content, and CD4^+^/CD8^+^ ratio in predicting the clinical features of CRC. We then combined the CD4^+^ and CD8^+^ T-lymphocyte content to build models and predict major clinical characteristics. We compared these models with the CD4^+^/CD8^+^ ratio to explore their advantages and disadvantages in predicting the clinical features of CRC.

**Discussion:**

Our results provide a theoretical basis for the future screening of effective markers in reflecting and predicting the progression of CRC. Changes in T lymphocyte subsets affect the progression of CRC to a certain extent, while their changes also reflect variations in the human immune system.

## Introduction

CRC is the third most common malignancy worldwide and has the second highest mortality rate (1). The morbidity and mortality rates continue to increase each year. Moreover, there is a trend toward a younger age in the incidence of CRC. Early diagnosis and treatment are paramount for malignant tumors; however, early-stage CRC often has no clinical symptoms. Moreover, in most cases, conventional screening tests, such as serum tumor marker tests, are not abnormal, which gives CRC the opportunity to infiltrate and grow further. When there are abnormalities in the relevant tests, CRC is considered to have reached an advanced stage. Even after effective surgical treatment, adjuvant chemotherapy, targeted therapy, and immunotherapy, there are varying degrees of recurrence and metastasis. Therefore, screening for valid and accurate markers that are closely associated with CRC to reflect and predict the progression of CRC is of great importance in current clinical management.

Tumor immunity has become a hot topic of research. Many researchers believe that tumor development is closely related to human immunity. The immune strength of the host can directly influence tumor development, and T lymphocytes play an indispensable role in the fight against tumors. T lymphocytes are classified into cytotoxic T cells and helper T cells according to the expression of cluster differentiation on the cell surface. The content of T lymphocytes subsets can reflect the level of immunity. CD4^+^ T lymphocytes can activate CD8^+^ T lymphocytes and promote the secretion of cytotoxic granules to kill tumor cells by modulating antigen presenting cells(APC) to provide stronger antigenic signals, or by providing co-stimulatory signals *via* dendritic cells (2, 3). Maintaining a dynamic balance in the content and ratio of CD4^+^ and CD8^+^ T lymphocytes is important for immune homeostasis, and any increase or decrease in the ratio of T lymphocytes can affect the immunity. Therefore, measuring the number of T-lymphocyte subsets in peripheral blood might predict tumor development and clinical features.

Previous studies have shown that the levels of CD4^+^ and CD8^+^ T lymphocytes are closely related to the clinical characteristics and prognosis of various malignancies, such as pancreatic cancer (4), bladder cancer (5), and breast cancer (6). However, studies on T-lymphocyte subset alterations in CRC are still scarce. In a clinical prognostic study of rectal cancer, Naito et al. (7) found that CD8^+^ T-lymphocyte content could be a valid independent indicator of CRC prognosis. Kuwahara et al. (8) predicted the prognosis of patients with CRC by integrating CD4^+^ and FOX3^+^ cells and concluded that a low proportion of CD4^+^ and FOX3^+^ cells suggests a poor prognosis. The studies mentioned above have shown the potential of changes in T-lymphocyte subsets to predict the prognosis and clinical features of CRC.

The present study specifically analyzed the association between changes in T-lymphocyte subsets and the clinical features of CRC. Furthermore, we predicted the level of development and malignancy of CRC based on changes in T-lymphocyte subsets. Ultimately, we combined CD4^+^ and CD8^+^ T-lymphocyte counts and used logistic regression to build models to better predict the major clinical characteristics of CRC.

## Materials and methods

### Selection of clinical patients

To reduce the influence of confounding factors and improve the accuracy of clinical trials, we formulated a series of criteria for patient screening. The inclusion criteria were as follows: (1) a preoperative cytological or pathological diagnosis of CRC; (2) no anti-tumor treatments after diagnosis of CRC; (3) no high-risk factors, such as CRC enterocutaneous fistula, intestinal obstruction, and gastrointestinal hemorrhage; (4) a Karnofsky score of >60; and (5) stable vital signs and normal consciousness. The exclusion criteria were as follows: (1) severe mental disorders; (2) use of immunosuppressive or immune-enhancing agents; (3) severe hematological disorders, autoimmune diseases, cardiovascular diseases, respiratory diseases, or sepsis; (4) pregnancy or breastfeeding; (5) allergies to biological products; (6) abnormal bone marrow function; (7) primary malignancy other than CRC; and (8) severe diabetes mellitus, hypertension, or obesity.

According to the above inclusion and exclusion criteria, postoperative pathology reports of 86 patients with pathologically confirmed CRC after standard laparoscopic resection at the China-Japan Union Hospital of Jilin University were collected from September 2021 to September 2022, with pathological staging according to the 8th American Joint Committee on Cancer criteria(AJCC). There were 55 male patients (mean age: 64.1 ± 10.8 years) and 31 female patients (mean age: 62.2 ± 9.8 years). The ethics committee approved the study, and all patients provided written informed consent. This study was conducted in accordance with the principles of the Declaration of Helsinki.

### Options for CRC surgery

To ensure consistency in the surgical procedure, we used standard minimally invasive laparoscopic surgery to resect colorectal malignancies. For malignant tumors of the right hemicolon, we used laparoscopic radical right hemicolectomy. In cases of malignant tumors of the left hemicolon, we used laparoscopic radical left hemicolectomy. For rectal cancer, we used either the laparoscopic Dixon or Miles procedure.

### Application of flow cytometry

To reduce the impact of surgical stress on immune function, we chose to collect peripheral blood on the day before surgery and on the tenth postoperative day. All patients had 200 μl of fresh peripheral blood drawn in the morning in the condition of limosis. CD4^+^ and CD8^+^ antibodies (40 μl each) were added within 4 h, mixed thoroughly, and protected from light for 30 min at room temperature. Red blood cell lysate (2.0 ml) was then added, mixed, and protected from light for 20 min at room temperature. After centrifugation (2,500 r/min for 5 min), the supernatant was removed, and 20 ml of 0.1% sodium azide phosphate buffer was added, mixed thoroughly, and centrifuged again (1,500 r/min for 5 min). CD4^+^ T-lymphocyte content, CD8^+^ T-lymphocyte content, and CD4^+^/CD8^+^ ratio were obtained by flow cytometry.

### Statistical analysis and data visualization

We used SPSS 22.0 software for statistical analysis and applied the Wilcoxon or Kruskal–Wallis rank-sum test to analyze the relationship between clinical pathological characteristics and T-lymphocyte subsets. The association between the main clinical features, T-lymphocyte subsets and the TNM stage was analyzed using the Chi-square test. The relationship between T-lymphocyte subsets and CEA was analyzed using Spearman's correlation test. For preoperative and postoperative changes in T-lymphocyte subsets, the paired Wilcoxon rank-sum test was used. The ggplot2 package of R software was then applied to visualize the results of the above data analysis and plot the combined comparison, scatter, and pairwise plots. The pROC package was used for the analysis of the receiver operating characteristic (ROC) curves of the independent and joint indicators, while the ggplot2 package was used to visualize the graphs. To facilitate a visual depiction of the relationships between the major clinical features of the 86 patients, we used the ggalluvial package to delineate alluvial plots.

### Efficacy analysis of sample size

To test the efficacy of this clinical trial, we used PASS software to analyze the statistical efficacy of the sample size required for the trial. The final estimated sample sizes are shown in Table 1. Table 1 shows that the vast majority of the clinical sample sizes that were collected were larger than the estimated sample sizes, which reflects the high statistical validity of our experiment.

**Table 1:** Estimated sample size required for each of the major clinical features.

Clinical characteristics	CD4^+^	CD8^+^	CD4^+^/CD8^+^ ratio
Ki67 expression	30	30	24
Organization differentiation	81	54	21
Clinical stage	51	30	18
T-stage	36	24	20
N-stage	42	32	27
CEA expression	118	50	61
Perineural invasion	50	84	52
Vascular invasion	88	26	30
Preoperative and postoperative trends	66	107	39

CEA, Carcinoembryonic antigen.

## Results

### General distribution of clinical pathological features of patients with CRC

**Figure 1:**
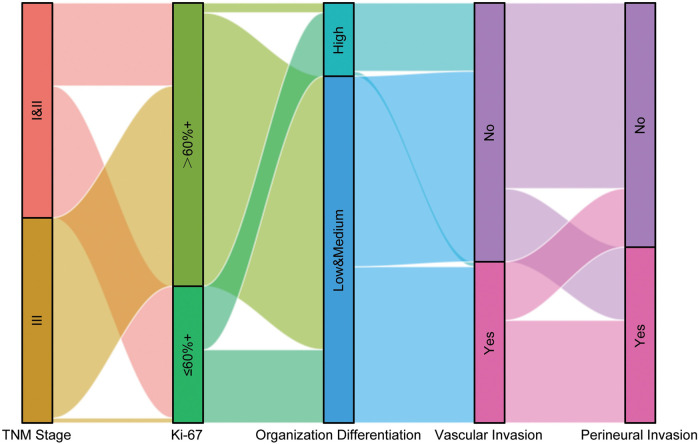
Overview of the distribution of clinical pathological features.

The alluvial plot visualizes the relationship between Ki67 expression, clinical pathological stage, degree of tumor differentiation, nerve invasion, and vascular invasion in all patients (Figure 1). The majority of patients with advanced colorectal cancer had high Ki67 expression. Correspondingly, a large proportion of patients with high Ki67 expression had poorly differentiated tumor tissue. Simultaneously, the majority of patients with nerve and vascular invasion had poorly differentiated tumors and a late clinical pathological stage, as well as high Ki67 expression. We applied the Chi-square test and presented the distribution of the main features of CRC patients in a clear and visual way in Table 2.

**Table 2:** The characteristics and T cell subsets in colorectal patients according to TNM stage.

Clinical Features	TNM Stage I & II	TNM stage III	Total	*P* Value
**Sex**				
Male	33	22	55	0.029
Female	11	20	31	
**Age (years)**				
≤65	21	21	42	0.83
>65	23	21	44	
**Tumor organization differentiation**				
High Differentiation	15	0	15	3.17 × 10^−8^
Medium Differentiation	25	15	40	
Low Differentiation	4	27	31	
**Tumor location**				
Right Hemicolon	15	10	25	0.11
Left Hemicolon	7	15	22	
Rectum	22	17	39	
**Ki-67 expression**				
≤60%+	27	1	28	5.38 × 10^−9^
>60%+	17	41	58	
**Vascular invasion**				
Yes	6	27	33	1.38 × 10^−6^
No	38	15	53	
**Perineural invasion**				
Yes	8	28	36	5.22 × 10^−6^
No	36	14	50	
**T-lymphocyte subsets**				
CD4^+^% > optimal cut-off value (38.5)	36	9	45	2.08 × 10^−8^
CD4^+^% < optimal cut-off value (38.5)	8	33	41	
CD8^+^% > optimal cut-off value (24.4)	6	39	45	1.94 × 10^−13^
CD8^+^% < optimal cut-off value (24.4)	38	3	41	
CD4^+^/CD8^+ ^> optimal cut-off value (1.625)	36	1	37	1.03 × 10^−13^
CD4^+^/CD8^+ ^< optimal cut-off value (1.625)	8	41	49	

The Chi-square test is applied in Table 2.

### Comparison of clinical pathological features

**Figure 2:**
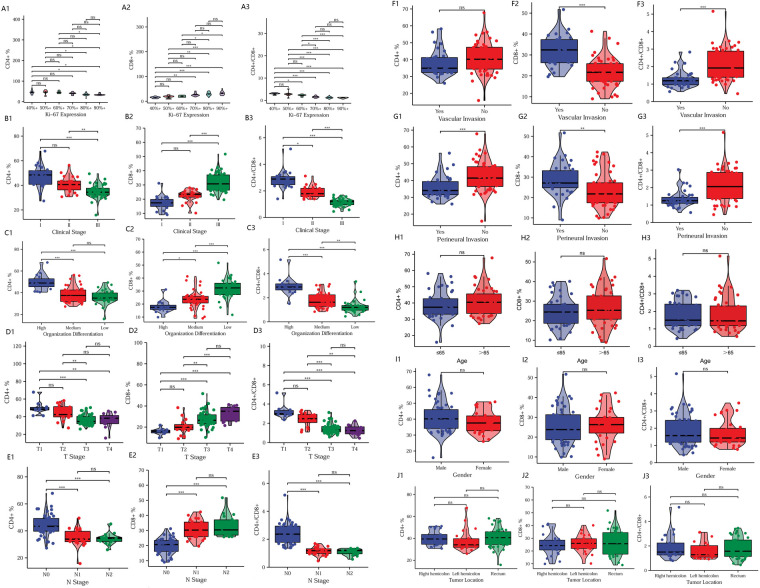
Comparison of clinical pathological features. Relationship between T-lymphocyte subsets and Ki67 expression (A1–A3), clinical pathological stage (B1–B3), tumor differentiation (C1–C3), T-stage (D1–D3), N-stage (E1–E3), vascular invasion (F1–F3), perineural invasion (G1–G3), age (H1–H3), sex (I1–I3), and tumor site (J1–J3). *P < 0.05; **P < 0.01; ***P < 0.001; ns, not significant.

We analyzed the differences in T-lymphocyte subsets between major clinical pathological features. As Ki67 expression increased, the CD4^+^ T-lymphocyte content and CD4^+^/CD8^+^ ratio gradually decreased, while CD8+ T-lymphocyte content continued to increase (Figures 2A1–3). With the increase in clinical pathological stage (Figures 2B1–3), T-stage (Figures 2D1–3), N-stage (Figures 2E1–3), vascular invasion (Figures 2F1–3), and perineural invasion (Figures 2G1–3); the decrease in tumor tissue differentiation (Figures 2C1–3), the CD4^+^ T-lymphocyte content and the CD4^+^/CD8^+^ ratio significantly decreased, while the CD8^+^ T-lymphocyte content significantly increased. There was no significant difference in T-lymphocyte subsets by age, sex, or tumor site (*P* > 0.05) (Figures 2H1–J3). The results suggest that T-lymphocyte subsets have strong association with major clinical pathological features. Thus, T lymphocyte subsets have the potential to fully reflect changes in clinical features.

### Correlation between CEA and T-lymphocyte subsets

**Figure 3:**
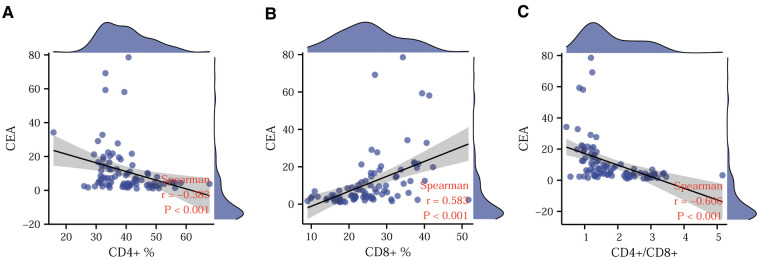
Correlation between CEA and T-lymphocyte subsets. Correlations of CD4^+^ (**A**), CD8^+^ (**B**), and CD4^+^/CD8^+^ ratio (**C**) with CEA. CEA. Carcinoembryonic antigen.

To further explore the correlation between CEA and T-lymphocyte subsets, we used Spearman's correlation analysis and depicted scatter plots. Figures 3A,C show a significant negative correlation of CD4^+^ T-lymphocyte content and CD4^+^/CD8^+^ ratio with CEA, while Figure 3B shows a significant positive correlation between CD8^+^ T-lymphocyte content and CEA. Based on the absolute magnitude of r, the CD4^+^/CD8^+^ ratio correlated most strongly with CEA, followed by CD8^+^ T-lymphocyte content and CD4^+^ T-lymphocyte content.

### Trends in preoperative and postoperative T-lymphocyte subsets

**Figure 4:**
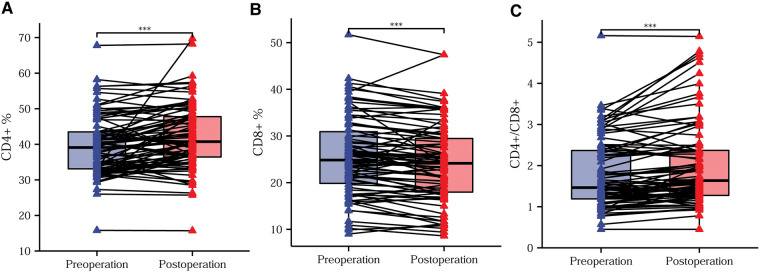
Trends in preoperative and postoperative T-lymphocyte subsets. Differences in the CD4^+^ T-lymphocyte content (**A**), CD8^+^ T-lymphocyte content (**B**), and CD4^+^/CD8^+^ ratio (**C**) before compared with after surgery. *P < 0.05; **P < 0.01; ***P < 0.001; ns, not significant.

In order to reduce the effect of confounding factors, such as surgical stress and the postoperative inflammatory response on postoperative immunity, we drew peripheral blood for flow cytometry analysis on the tenth postoperative day after normalization of leukocytes and neutrophils. Postoperatively, we routinely administered anti-inflammatory, acid-suppressive, analgesic, and anti-emetic medications, and we did not administer immunosuppressive or immune-enhancing agents. After effective treatment with laparoscopic surgery, the CD4^+^ T-lymphocyte content and the CD4^+^/CD8^+^ ratio increased significantly, while the CD8^+^ T-lymphocyte content decreased significantly (Figures 4A–C).

### Prediction of different clinical features by T-lymphocyte subset

**Figure 5:**
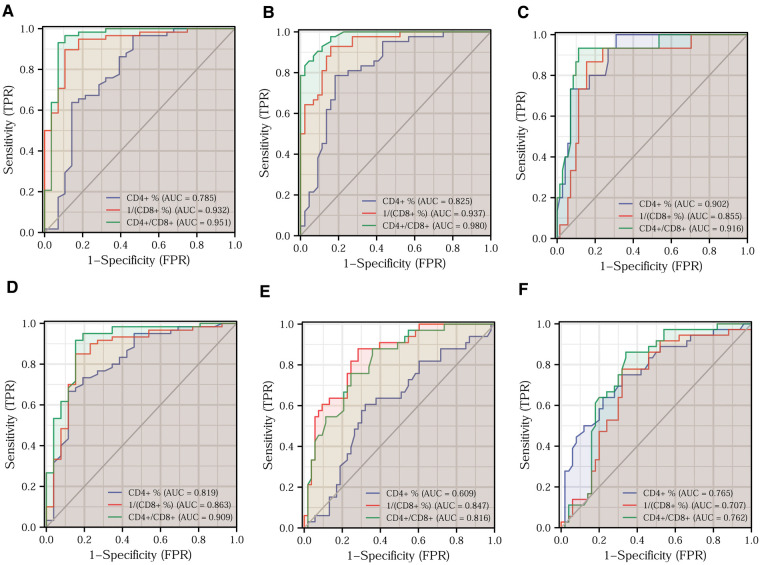
Prediction of different clinical features by T-lymphocyte subset. Efficacy of T-lymphocyte subsets for predicting Ki67 expression (**A**), clinical pathological stage (**B**), tumor differentiation (**C**), T-stage (**D**), vascular invasion (**E**), and perineural invasion (**F**).

For testing the ability of T-lymphocyte subsets to predict clinical features, we plotted ROC curves to judge the predictive performance of different indicators and found the best cut-off value. The DeLong test was then applied to identify significant differences between the predictive merits of these indicators. Based on the fact that a Ki67 positivity of 60% is often used as the dividing line in daily clinical practice, we classified Ki67 positivity of ≤60% as low expression and Ki67 positivity of >60% as high expression. A comparison of the predictive efficacy of T-lymphocyte subsets is depicted in the ROC curves in Figure 5A. All three indicators had high predictive performance. The difference in predictive performance between the CD4^+^/CD8^+^ ratio and 1/CD8^+^ was not statistically significant. Based on the situation of lymph node metastasis, we classified clinical pathological stages I and II as early colorectal cancer and stage III as advanced colorectal cancer. Using the DeLong test, we found the best predictive efficacy for the CD4^+^/CD8^+^ ratio (Figure 5B). We then divided the moderately and poorly differentiated adenocarcinomas into one group and the highly differentiated adenocarcinomas into another. Figure 5C clearly shows that the CD4^+^/CD8^+^ ratio had the best predictive performance. We further classified T1 and T2 as the low tumor infiltration group, T3 and T4 as the high tumor infiltration group (Figure 5D). After applying the DeLong test, the CD4^+^/CD8^+^ ratio continued to have the best predictive performance. Comparisons of the predictive efficacy of the T-lymphocyte subsets for vascular invasion and perineural invasion are shown in Figures 5E,F. Finally, we tabulated the best cut-off values and corresponding sensitivity, specificity of the three indicators for the prediction of major clinical features (Tables 3, 4).

**Table 3:** Optimal cut-off values for T-lymphocyte subsets to predict the major clinical features of colorectal cancer.

Clinical characteristics	CD4^+^ (%)	1/(CD8^+^ %)	CD4^+^/CD8^+^ ratio
Ki67 expression	47.45	0.046	1.895
Clinical stage	38.50	0.041	1.625
Organization differentiation	40.45	0.048	2.365
T-stage	39.10	0.046	2.215
Vascular invasion	37.05	0.040	1.625
Perineural invasion	39.10	0.040	1.625

**Table 4:** Sensitivity and specificity of the best Cut-off values of T-lymphocyte subsets.

Clinical characteristics	CD4^+^ (%)		1/(CD8^+^ %)		CD4^+^/CD8^+^ ratio	
	Sensitivity	Specificity	Sensitivity	Specificity	Sensitivity	Specificity
Ki-67 expression	96.6%	53.6%	89.7%	89.3%	93.1%	92.9%
Clinical stage	78.6%	81.8%	92.9%	84.1%	97.6%	84.1%
Organization differentiation	98.6%	69.0%	86.7%	84.5%	93.3%	88.7%
T stage	66.7%	88.5%	85.0%	84.6%	91.7%	84.6%
Vascular invasion	60.6%	67.9%	87.9%	71.7%	87.9%	64.2%
Perineural invasion	75.0%	68.0%	77.8%	68.0%	86.1%	66.0%

### Model indicators predicting different clinical features

**Figure 6:**
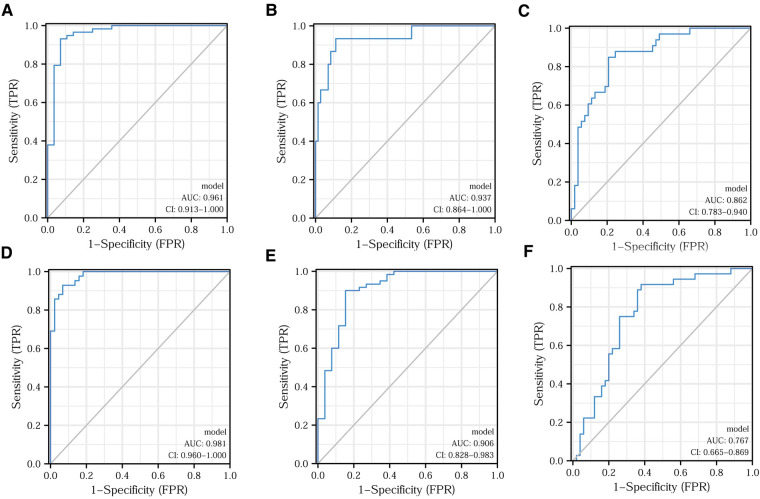
Model indicators predicting different clinical features. Efficacy of model indicators for predicting Ki67 expression (**A**), tumor differentiation (**B**), vascular infiltration (**C**), clinical pathological stage (**D**), T-stage (**E**), and nerve invasion (**F**).

Applying the logistic regression analysis, we combined CD4^+^ and CD8^+^ T lymphocytes to build models for various clinical characteristics to identify more accurate indicators. Figures 6A–C shows the predictive efficacy of the model indicators for Ki67 expression, tumor differentiation, and vascular invasion in the form of ROC curves. After applying the DeLong test, we found that the predictive efficacy of the model indicators was better than that of the CD4^+^/CD8^+^ ratio. The model indicators predicting clinical pathological stage, T-stage, and perineural invasion are depicted in Figures 6D–F in the forms of ROC. The model indicators did not show better predictive efficacy than the CD4^+^/CD8^+^ ratio after applying the DeLong test. The relevant data for each model indicator are presented in Table 5.

**Table 5:** Predictive model indicators for each clinical characteristic.

Clinical Characteristics	Model Indicators	The Best Cut-off Value	Sensitivity	Specificity
Ki-67 expression	− 2.1488 − 0.1598 × CD4^+^ + 0.4284 × CD8^+^	0.608	93.1%	92.9%
Organization differentiation	− 5.7692 + 0.2147 × CD4^+^ − 0.2488 × CD8^+^	− 1.254	93.3%	88.7%
Vascular invasion	− 7.2618 + 0.0244 × CD4^+^ + 0.2202 × CD8^+^	− 0.749	84.8%	79.2%
Clinical stage	− 1.5199 − 0.3531 × CD4^+^ + 0.5897 × CD8^+^	0.409	92.9%	93.2%
T stage	2.1721 − 0.1509 × CD4^+^ + 0.2161 × CD8^+^	0.121	90.0%	84.6%
Perineural invasion	2.3227 − 0.1043 × CD4^+^ + 0.0538 × CD8^+^	− 0.588	91.7%	62.0%

## Discussion

CRC development is a multifactorial, multistep process involving a wide range of mechanisms. Tumor immune escape, low body immune surveillance, and an altered tumor microenvironment are all involved (9). During the body's anti-tumor process, T lymphocytes kill tumor cells at the primary site and reduce the risk of tumor spread and distant metastasis. However, when cytotoxic T cells and helper T cells are hypofunctional, the release of cytotoxic granules is significantly reduced. As a result, the immune response to tumor antigens decreases (10). Thus, T-lymphocyte subsets in peripheral blood might have association with progression of malignant tumor, and as such, they have the potential to reflect and predict major clinical pathological characteristics of CRC.

The results of this study suggest that T-lymphocyte subsets in peripheral blood are closely associated with the major clinical features of CRC. Increased expression of Ki67, which a proliferating cell-associated antigen, implies a strong proliferative capacity of tumor cells and a poorer prognosis for patients (11). We found that CD4^+^ T-lymphocyte content and CD4^+^/CD8^+^ ratio decreased with an increase in Ki67 expression, while CD8^+^ T-lymphocyte content gradually increased. This means that the decrease in CD4^+^ T-lymphocyte content and CD4^+^/CD8^+^ ratio suggest a progressive state and accelerated proliferation of CRC. A high CD8^+^ T-lymphocyte content may be associated with poor clinical outcomes. Clinical stage is a comprehensive evaluation of malignancy, and an advanced stage equates to a higher probability of tumor recurrence and metastasis after surgery (12). In this study, CD4^+^ T-lymphocyte content and CD4^+^/CD8^+^ ratio were significantly negatively correlated with clinical stage, while CD8^+^ T-lymphocyte content was significantly positively correlated with clinical stage. This implies that T-lymphocyte subsets are predictive of clinical stage. An increase in CD4^+^ T-lymphocyte content and CD4^+^/CD8^+^ ratio means that the tumor is still in an early clinical stage, while an increase in CD8^+^ T-lymphocyte content means that the tumor has escalated to an advanced stage. The degree of tumor differentiation is often an important risk factor that affects the prognosis of patients (13). Our results found significant correlations of reduced CD4^+^ T-lymphocyte content, reduced CD4^+^/CD8^+^ ratio, and increased CD8^+^ T-lymphocyte content with poorer tumor tissue differentiation. This further illustrates that the alteration in T-lymphocyte subsets influences tumor progression.

It is known that a later T-stage represents deeper tumor tissue infiltration, while a later N-stage suggests a higher number of lymph node metastases. The progression of both stages suggests a worrying outcome for patient survival (14). The changes in T-lymphocyte subsets all showed similar trends when combined with clinical stage, T-stage, and N-stage in our current study. This implies that a decrease in CD4^+^ T-lymphocyte content, a decrease in CD4^+^/CD8^+^ ratio, and an increase in CD8^+^ T-lymphocyte content indicate disappointing clinical pathological features.

Vascular and nerve invasion, as high-risk factors for CRC, imply an increased risk of distant tumor metastasis. Our findings clearly demonstrate that a decrease in CD4^+^ T-lymphocyte content, a decrease in CD4^+^/CD8^+^ ratio, and an increase in CD8^+^ T-lymphocyte content may increase the risk of vascular and nerve invasion of CRC. As one of the most commonly used tumor markers that is associated with CRC in clinical practice, although the sensitivity and specificity of CEA are not ideal, to a certain degree, increase of CEA can reflect the progression, postoperative recurrence and metastasis of CRC (15). Spearman's correlation analysis showed that all T-lymphocyte subsets were significantly correlated with CEA. This further proves that a decrease in CD4^+^ T-lymphocyte content and CD4^+^/CD8^+^ ratio and an increase in CD8^+^ T-lymphocyte content are closely associated with a desperate clinical profile and an unpromising outcome. Conversely, T-lymphocyte subsets were unrelated to age, sex and tumor site. Taken together, these results suggest that T-lymphocyte subsets have a high degree of confidence in reflecting the clinical features of CRC.

The preoperative and postoperative changes in T-lymphocyte subsets suggest that the body's immune response was enhanced after the primary malignant lesion was removed by effective surgical intervention. Malignant tumors and the immune system are always in a process of resistance. The greater the tumor malignancy, the more aggressive it is and the stronger its suppressive effect on the body's immunity, with a consequent decrease in immune function. When the malignant tumor was eradicated, the suppression of immunity vanished and immune function gradually returned to normal. Therefore, alterations in T-lymphocyte subsets have the potential to be used in postoperative monitoring of tumor metastasis and recurrence, as well as in evaluating drug efficacy. Further analysis can be formulated by tracking the patient's postoperative survival.

As effective tumor markers should have both high sensitivity and specificity, we analyzed and compared the predictive power of the T-lymphocyte subsets for different clinical features. Through the ROC curve analysis, we clearly discerned that the CD4^+^ and CD8^+^ T-lymphocyte content and CD4^+^/CD8^+^ ratio have high sensitivity and specificity. Therefore, the above analysis of the relationship between T-lymphocyte subsets and major clinical features suggests that, as immune indicators, CD4^+^ and CD8^+^ T-lymphocyte content and CD4^+^/CD8^+^ ratio are significant predictors of the clinical features of CRC. The decrease in CD4^+^ T-lymphocyte content and CD4^+^/CD8^+^ ratio and the increase in CD8^+^ T-lymphocyte content reflect a tendency for immunity to decline, leading to the development of colorectal malignancies. The lower the body's immune defense, the more rapid the development of malignant tumors, the higher the degree of malignancy, and the more worrisome the clinicopathological features of the patients. In summary, the decrease in CD4^+^ T-lymphocyte content and CD4^+^/CD8^+^ ratio and the increase in CD8^+^ T-lymphocyte content are indicative of a worrying clinicopathological outcome and indirectly suggest an unpromising prognostic outcome for patients.

CD4^+^ and CD8^+^ T-lymphocyte content have their own advantages and disadvantages for predicting different clinical features. The predictive efficacy of the CD4^+^/CD8^+^ ratio for predicting major clinical features was superior to each of CD4^+^ and CD8^+^ T-lymphocyte content alone. After combining CD4^+^ and CD8^+^ T-lymphocyte content, we attempted to identify model indicators with higher sensitivity and specificity in predicting clinical features by applying logistic regression. Overall, the predictive efficacy of our constructed model indicators was superior to CD4^+^ and CD8^+^ T-lymphocyte content alone. Compared with the CD4^+^/CD8^+^ ratio, the model indicators had certain advantages. For example, model indicators appear to be more effective than the CD4^+^/CD8^+^ ratio for predicting Ki67 expression, tumor differentiation and vascular invasion. The model indicators and CD4^+^/CD8^+^ ratio were all suitable for predicting clinical pathological stage, T-stage, and nerve invasion. In the future, we will optimize and validate the model indicators using more eligible samples.

Collectively, T lymphocytes play an essential role in the fight against tumor cell invasion, metastasis, and recurrence (16). However, different T-lymphocyte subsets play different roles in the immune defense (17). CD4^+^ T cells activate macrophages and CD8^+^ T cells by secreting cytokines, such as interleukin (IL)-2, IL-12, and interferon-γ. We suggest that when malignancies weaken the body's immunity through different immunosuppressive pathways, the CD4^+^ T-lymphocyte content may decrease as the body's immune function is weakened. Consequently, the immune-mediating role of CD4^+^ T lymphocytes also decreases. Thus, a low CD4^+^ T-lymphocyte content is associated with worrying clinical features in the course of tumor development. Thompson et al. demonstrates that both in gastrointestinal tumor tissue and in the peritumor stroma, the higher the density of CD8^+^ T lymphocyte infiltration, the lower the progression-free survival and overall survival of patients (18). We believe that the anti-tumor effects of cytotoxic T cells that release granzyme and perforin (3) are substantially undermined as tumor malignancy increases. Immune dysfunction and immune rejection of CD8^+^ T-lymphocyte was used to evaluate and assess prognosis of various malignant tumors (19). The more severe immune dysfunction and immune rejection of CD8^+^ T-lymphocyte is, the more unpromising outcome of malignant tumor will be. It has been shown that a decrease in the level of immune-responsive CD8^+^ T lymphocytes is a key factor in the progression of CRC (20). The CD8^+^ T lymphocytes can be further divided into CD8^+^CD28^+^ T lymphocytes (CTL) and CD8^+^CD28^−^ T lymphocytes based on the expression of CD28 on the surface of CD8^+^ T lymphocytes, of which CD8^+^CD28^−^ T lymphocytes are a class of regulatory T cells that do not have tumor-killing functions. Some researchers suggest that the chronic stimulation of cancer antigens leads to the cycle activation of the immune cells, which eventually causes CD8^+^CD28^−^ T lymphocytes to proliferate and exert negative regulatory functions, thus inhibiting the tumor-killing effect of CTL (21). At the same time, tumor cells produce large amounts of enzymes that degrade arginine and tryptophan to compete with immune cells for oxygen and nutrients, which ultimately leads to loss of immune function of CD8^+^ T lymphocytes (22). Furthermore, some researchers have suggested that CD8^+^ T cell inactivation originates from T cell exhaustion. In their study they found that naive CD8^+^ T cells targeting tumor antigens are first initiated in peripheral lymphoid tissues to generate stem cell-like PD-1^lo^CD8^+^ T cells with self-renewal properties, which migrate toward TME and form immune-responsive PD-1^lo^CD8^+^ T cells in response to chemokines CCL5 and CXCL9. However, in TME, due to continuous antigenic stimulation, stem cell-like PD-1^lo^CD8^+^ T cells differentiate and proliferate into substantial PD-1^hi^CD8^+^ T cells without immune function (23). Based on those theories, we speculate that although CD8^+^ T-lymphocyte content was higher in cases of CRC with advanced stage, poorer differentiation, and higher Ki67 expression, significantly fewer CD8^+^ T lymphocytes actually exerted anti-tumor effects because of the continuous cancer antigen stimulation, transformation of CD8^+^ T-cell subtypes, serious immune dysfunction and rejection. As a result, CD8^+^ T lymphocytes proliferate and infiltrate but lose their function in such tumor microenvironments. The majority of the remainder were compensated functional suppressed cytotoxic T cells. Overall, our results suggest that an increase in the CD4^+^/CD8^+^ ratio is associated with an increased immune response and may inhibit tumor progression. Conversely, a decrease in the CD4^+^/CD8^+^ ratio may be associated with a restricted immune response, allowing the tumor to proliferate.

## Conclusions

In summary, CD4^+^ T-lymphocyte content, CD8^+^ T-lymphocyte content, and CD4^+^/CD8^+^ ratio are useful predictors of clinical features in CRC. Alterations in these indicators are closely associated with the clinical features and surgical treatment of CRC. A decreased CD4^+^ T-lymphocyte content, a decreased CD4^+^/CD8^+^ ratio, and an increased CD8^+^ T-lymphocyte content are associated with a poorer CRC prognosis. As a result, CD4^+^ and CD8^+^ T-lymphocyte content and CD4^+^/CD8^+^ ratio are expected to suitably reflect and predict major clinical characteristics of CRC. These results have potential clinical significance for reflecting and predicting the progression and outcome of CRC in the future.

## Data Availability

The original contributions presented in the study are included in the article, further inquiries can be directed to the corresponding authors.
